# Impact of Microbes on the Pathogenesis of Primary Biliary Cirrhosis (PBC) and Primary Sclerosing Cholangitis (PSC)

**DOI:** 10.3390/ijms17111864

**Published:** 2016-11-09

**Authors:** Jochen Mattner

**Affiliations:** Mikrobiologisches Institut—Klinische Mikrobiologie, Immunologie und Hygiene, Universitätsklinikum Erlangen and Friedrich-Alexander Universität (FAU) Erlangen-Nürnberg, Wasserturmstr. 3/5, D-91054 Erlangen, Germany; jochen.mattner@uk-erlangen.de; Tel.: +49-9131-85-23640; Fax: +49-9131-85-1001

**Keywords:** PSC, PBC, intestinal microbiota, bile acids

## Abstract

Primary biliary cirrhosis (PBC) and primary sclerosing cholangitis (PSC) represent the major clinical entities of chronic cholestatic liver diseases. Both disorders are characterized by portal inflammation and slowly progress to obliterative fibrosis and eventually liver cirrhosis. Although immune-pathogenic mechanisms have been implicated in the pathogenesis of PBC and PSC, neither disorder is considered to be a classical autoimmune disease, as PSC and PBC patients do not respond to immune-suppressants. Furthermore, the decreased bile flow resulting from the immune-mediated tissue assault and the subsequent accumulation of toxic bile products in PBC and PSC not only perpetuates biliary epithelial damage, but also alters the composition of the intestinal and biliary microbiota and its mutual interactions with the host. Consistent with the close association of PSC and inflammatory bowel disease (IBD), the polyclonal hyper IgM response in PBC and (auto-)antibodies which cross-react to microbial antigens in both diseases, an expansion of individual microbes leads to shifts in the composition of the intestinal or biliary microbiota and a subsequent altered integrity of epithelial layers, promoting microbial translocation. These changes have been implicated in the pathogenesis of both devastating disorders. Thus, we will discuss here these recent findings in the context of novel and alternative therapeutic options.

## 1. Introduction

The liver commands several central metabolic and synthetic pathways in the body. Among these is also the regulation of bile (acid) metabolism [1].

Bile is synthesized in the pericentral hepatocytes, drained through the bile ducts into the hepatic duct, and released into the duodenum by the sphincter of Oddi [2]. Bile acids—which constitute for half of the organic biliary compounds [2,3]—are conserved under physiologic conditions; about 95% of bile acids are reabsorbed by the intestinal epithelium and transported back through the portal vein system to the liver, where they are re-conjugated and re-secreted into the bile fluid (enterohepatic circulation) [4].

Bile acids themselves exhibit several fundamentally important functions [2,5]. These include the elimination of many waste products and catabolites such as cholesterol and bilirubin from the body via the feces, the adsorption and digestion of lipids and fat-soluble vitamins in the gut, and—together with immunoglobulin A (IgA) (which is secreted from the epithelium into the bile fluid)—potent anti-microbial properties that inhibit bacterial growth and adhesion, thus protecting against ascending infections within the biliary tract. However, as there exist multiple microbes which are tolerant against bile [3], the anti-microbial effects of bile (acids) selectively restrain certain microbial species, and subsequently affect the composition of the complete intestinal or biliary microflora. Thus, an obstruction of the bile flow as it occurs in primary biliary cirrhosis (PBC) and primary sclerosing cholangitis (PSC) alters the microbiota, the susceptibility to infection, and the integrity of the epithelial layers [6,7,8]. As the liver—due to its anatomic location within the blood circulation—is exposed to various nutritional and microbial compounds from the gastrointestinal tract, a metabolic and/or microbial distortion might promote inappropriate inflammatory immune reactions within hepatic tissues.

## 2. Mutual Interactions between the Bile and the Intestinal Microbiota

The diverse and complex microbial community within the gastrointestinal tract produces metabolites and extracts nutrients from a large range of molecules that enzymes of the host are unable to convert [9]. Many of these nutrients and metabolites derived from the commensal microbiota have been implicated in the development, homeostasis, and function of the immune system, suggesting that microbial commensals influence host immunity via nutrient- and metabolite-dependent mechanisms [10]. Novel findings suggested that—similar to the situation in the intestine—the gallbladder also harbors a complex microbiota, and that the biliary mucosa features a chemical, mechanical, and immunological barrier, ensuring immunological tolerance against microbial commensals [6].

The regulation of the bile acid pool is one example of the interference of the microbial metabolism with the host [11]. The microbiota can modify the remaining 5% of the total bile acid pool which are not absorbed by the epithelium in multiple ways [12]. The modifications of bile acids retained in the gut depend on the intestinal and biliary bile acid flow, the population of the respective mucosal tissues with bile-converting microbes, and the properties and activity of the responsible microbial enzymes [13]. The primary bile acids—cholic and chenodeoxycholic acid, for example—can be converted to more than 20 different secondary or tertiary bile acid metabolites by a particular subset of anaerobic bacteria in the gut [9,14]. For example, *Ruminococcus* can form ursodeoxycholic acid (UDCA) [15], a tertiary bile acid which is the only FDA-approved drug in the treatment of PBC. Furthermore, bacterial bile salt hydrolases (BSH), abundant enzymes found in all major bacterial phyla [16], deconjugate primary bile acids such as glycocholate or taurocholate to cholate, and profoundly alter both local, gastrointestinal, and systemic hepatic host functions; thus, gastrointestinal BSH expression results in local bile acid deconjugation with concomitant alterations in lipid and cholesterol metabolism, signaling functions, and weight gain [3,17,18,19,20]. On the other hand, the microbiota might metabolize the deliberated amino acids from deconjugation as an energy or metabolic source and/or increase their survival or tolerance to bile [3,21,22]. Both cholesterol and lipid metabolism are affected in PBC and PSC, resulting in vitamin deficiencies, distortions in bile acids, and perpetuation of biliary disease [23,24,25,26,27]. Probiotics have been suggested to increase bile acid synthesis and metabolism in humans and mice [28,29], and might therefore interfere with the described phenotypes, although further studies are required to delineate the distinct effects.

Conversely, bile acids control bacteria [30], exert anti-microbial properties [31], and thus modulate the microbiota both directly and indirectly through the activation of innate immune genes [32]. The loss of secondary bile acids, for example, has been associated with susceptibility to infection by pathogenic bacteria, and a restoration of the secondary bile acid pool promotes colonization resistance [33]. The decreased bile acid secretion in liver cirrhosis is associated with bacterial overgrowth in the gut [34,35]. Bile duct ligation also promotes bacterial proliferation and replication [36,37]. Along with the suppression of bacterial expansion in vivo, bile—predominantly the unconjugated bile acids therein—inhibit bacterial growth in vitro [3,38]. Long chain fatty acids (which are associated with bile acids in mixed micelles) likely contribute to the antimicrobial effects of bile fluid [39,40,41]. However, there exist several pathogenic microbial species which are tolerant against bile, such as *Escherichia coli* or *Helicobacter* spp. [42,43,44,45,46]. Furthermore, the composition of the bile fluid might be altered in PSC and PBC, allowing unusual bacteria to expand and/or even perpetuate ascending infections within the biliary tree. Thus, host metabolism can be affected through microbial modifications of bile acids, which lead to altered immune signaling via bile acid receptors, but also modified immune responses triggered by an altered microbiota composition. Further studies are needed to expand on these ideas.

## 3. Association of Distinct Bacteria with Primary Sclerosing Cholangitis (PSC) and Primary Biliary Cirrhosis (PBC)

There exist several indirect hints that microbes are involved in the pathogenesis of PBC and PSC: a polyclonal IgM response in PBC [47,48,49], which can be frequently observed during chronic infections; an increased risk of patients with recurrent urinary tract infections to develop PBC [50,51,52,53,54,55]; and the close association of PSC with inflammatory bowel disease (IBD), particularly ulcerative colitis (UC) [56,57].

More direct hints include the linkage of different bacteria and viruses to the pathogenesis of PBC [58,59,60,61,62,63] and PSC [64,65]. Molecular mimicry has been proposed as one potential pathogenic mechanism underlying immune-mediated biliary damage. Thus, antibodies in the sera of PBC patients which bind to the mitochondrial E2 subunit of the pyruvate dehydrogenase complex (PDC-E2)—the signature antigen of PBC—also cross-react to conserved bacterial proteins [66,67,68,69,70,71,72,73]. These include the ATP (adenosine triphosphate)-dependent Clp protease of *Escherichia coli*, the β-galactosidase of *Lactobacillus delbrueckii* (a constituent of the vaginal flora), and two yet-undefined lipoylated proteins of *Novosphingobium aromaticivorans*, a ubiquitous α-proteobacterium also found at mucosal surfaces and in the feces of humans [66,67,68,69,70,71,74,75,76]. Furthermore, the infection of genetically susceptible mouse strains with *Novosphingobium aromaticivorans* induced anti-PDC E2 responses and liver lesions resembling PBC in humans [77,78,79]. *Novosphingobium* spp. can also metabolize xenobiotics [80,81,82], and thus interfere with enterohepatic bile acid cycling and hormone metabolism. All these characteristics and metabolic interactions might contribute to the break of self-tolerance within the unique immunological milieu of the liver [83]. As anti-PDC-E2 antibody responses precede the induction of liver pathology [84], the period between the detection of antibody responses and the onset of biliary pathology may mark a time frame in which an application of antibiotics may halt the development of full-blown PBC, assuming that the underlying pathogenic mechanisms are triggered by a bacterial infection. Furthermore, a microbial-mediated insult or a tissue-tropism of distinct microbes with homology to PDC-E2 might underlie the immune attack against biliary epithelial cells. Thus, immune responses against PDC-E2 might primarily target a microbe and not a mitochondrial enzyme. This would explain the tissue-specific pathology in PBC, although the hallmark antigen of PBC is ubiquitously expressed.

Bacteria have also been linked to the pathogenesis of PSC [64,65]. Given that the intestine is a key regulator of immunopathogenic T helper type 17 (Th17) cells [85], and that increased Th17 responses to pathogen stimulation in PSC patients have been reported [86], this may underpin a common disease mechanism of PSC and IBD, and open up novel treatment avenues based on rational targeting of immune pathways. Mucosal lymphocytes may also play a pivotal role in graft versus host disease affecting the liver, and there is increasing evidence to support dysregulated mucosal immunity as being responsible for the hepatic manifestations of gluten-sensitive enteropathy, graft versus host disease, as wells as the pancreatobiliary manifestations of IgG4-related disease [85].

## 4. Changes in the Composition of the Microbiota in PBC and PSC Patients

Compared to the intestine, the microbiota of the biliary tract contains relatively lower levels of *Bacteroidetes*, but comparable amounts of *Firmicutes*. In contrast, the relative numbers of *Proteobacteria*, *TM7*, *Tenericutes*, *Actinobacteria*, and *Cyanobacteria* are increased [6,87]. However, as bile samples in these studies are frequently collected via endoscopic retrograde cholangio-pancreatography (ERCP), contaminations with the intestinal microbiota are likely.

Specific alterations of the biliary microbiota have been reported in PBC patients undergoing liver transplantation compared to patients undergoing cholecystectomy because of gastric cancer or cholecystolithiasis, or an explanation for donor liver transplants [88]. The 16S-RNA-based analysis revealed an accumulation of Gram-positive cocci in the bile of PBC patients with end-stage disease, whereas Gram-negative bacteria were predominantly recovered from patients with cholecystolithiasis. In contrast, bacteria were detected just in 1 out of 12 patients who did not suffer from primary hepatobiliary disease. Thus, this study contrasts with an analysis of healthy pigs, in which a complex biliary microbiota was detected in all analyzed animals [89]. These discrepancies, as well as the question of whether changes in the biliary microbiota are functionally linked to the development and/or progression of PBC, remain to be resolved.

Dysbiosis has been extensively associated with the pathogenesis of IBD [90,91]. Thus, as PSC is frequently accompanied by IBD [56,57], changes in the microbiota are presumably also involved in the pathogenesis of PSC. In this context, it has been speculated that a putative gut-derived microbial trigger reaches the biliary epithelium via the leaky, inflamed intestinal epithelium, and the enterohepatic circulation [92]. Indeed, an experimentally-induced expansion of bacteria in the small bowel of rats perpetuated hepatobiliary inflammation resembling histological and cholangiographic features of PSC [93]. The application of a peptidoglycan-degrading enzyme improves biliary pathology in these rats, suggesting that microbe-associated molecular patterns are indeed involved in the induction of biliary disease [94]. Clinical studies describing a stabilization of liver enzymes upon antibiotic treatment further support the hypothesis that microbial triggers might be involved in the induction and/or progression of PSC [92]. In addition, alterations in the intestinal microbiota have been described in this patient cohort. For example, the relative abundance of *Clostridiales* II and the overall bacterial diversity is lower in PSC patients compared to UC patients (without concomitant PSC) and control individuals [95]. Interestingly, it has been suggested in this context that PSC-dependent dysbiosis is distinct and independent from the dysbiosis observed in IBD [96,97]. Furthermore, infections can complicate the course of disease. Thus, it has been reported that persistent biliary candidiasis is associated with a markedly reduced transplantation-free survival of PSC patients [98].

A recent report links a dysfunctional fucusyltransferase-2 (FUT2) to PSC [87] and Crohn disease (CD) [99], and the FUT2 genotype to the altered composition of the microbiota in the intestine and bile [87,99]. A genetic polymorphism within FUT2 alters the fucosylation (a form of glycosylation) of proteins which are shed into the intestinal [100] and biliary lumen [87]. Fucosylated proteins are known to influence metabolic pathways of intestinal commensals. Thus, they reduce (for example) the expression of bacterial virulence genes [100], and an increased abundance of *Firmicutes* as well as relatively lower levels of *Proteobacteria* and an overall reduced species richness in the bile have been associated with dysfunctional FUT2 [87]. Altogether, although compelling evidence for specific microbes associated with PSC is still lacking, these examples strongly suggest that microbial triggers influence PSC progression and that the microbiota can induce clinical complications in PSC patients.

Experimental evidence for the association of microbes with the pathogenesis of PSC is provided by additional studies. The (auto-)antibodies detected in PSC patients include atypical perinuclear antineutrophil cytoplasmic antibodies (p-ANCAs). p-ANCAs bind to the autoantigen β-tubulin isotype 5 (TBB-5) and its bacterial homologue FtsZ, which is ubiquitously expressed among the commensal microbiota [101]. p-ANCA, anti-TBB-5, and anti-FtsZ antibodies could only be detected in IL-10-deficient mice kept under single pathogen-free, but not germ-free condition [101]. Furthermore, the attachment of autoantibodies to biliary epithelial cells triggered an upregulation of toll-like receptor (TLR) expression, which might sensitize the biliary tract to microbial signals [102]. Thus, microbial triggers likely contribute to the formation and pathogenicity of these auto-antibodies.

However, the microbiota might also protect from PSC. In this context, it has been recently shown that fibrosis, ductular reaction, and ductopenia are significantly more severe in germ-free (GF) mutltidrug resistant 2 knockout (mdr2^−/−^) mice than in conventionally housed animals [103]. In this context, it is interesting to note that germ-free animals retain a global pool of only primary bile acids [104].

## 5. Conclusions

There exists compelling evidence that microbial agents and/or changes in the intestinal/biliary microbiota are involved in the pathogenesis of PBC and PSC. Thus, the application of antibiotics might be a novel and alternative therapeutic option for the treatment of these devastating diseases. Indeed, the application of antibiotics such as minocycline, metronidazole, or vancomycin has been reported to improve liver biochemistry and clinical symptoms of PSC patients, whereas the effects on Mayo risk scores and liver histology were less significant [92,105,106,107,108,109,110,111]. However, as antibiotics [112,113] are not species-specific, and both diseases are associated with alterations in the composition of the microbiota, the choice of the antibiotic substance, the duration of the use, the bile-permeability, and the availability have to be carefully considered, especially with respect to the fact that many of the microbial species and their function for the maintenance of immune tolerance have not yet been elucidated. Thus, antibiotic therapy appears to be promising. However, a long-term antibiotic can be associated with several adverse effects [114,115,116], including the perturbation of the resident microflora, the selection and spread of resistant microorganisms, and the development of other pathologies, such as diarrhea, colitis, sepsis, inflammatory bowel disease, obesity, and allergies [117,118,119,120,121,122,123,124,125,126,127,128,129]. As pathogenic bacteria might use the biliary tree as route for an ascending infection, and as ursodeoxycholic acid (UCDA, one of the secondary bile acids) is the only FDA-approved drug currently available for the treatment of PBC, its mechanism of action might include anti-microbial effects, as recently shown for *Clostridium difficile* infections [130] and/or the correction of alterations within the composition of the intestinal and/or biliary microbiota [131,132]. In order to address these pending questions, clinical studies must be initiated, and the therapeutic regimen carefully considered in order to identify therapeutic targets and approaches that can be used to guide the development of effective therapies for PBC and PSC, but also allows the identification of common targets in immune-mediated disease for clinical intervention in the future.

## References

[1] Russell D.W. (2003). The enzymes, regulation, and genetics of bile acid synthesis. Annu. Rev. Biochem..

[2] Hofmann A.F. (1999). Bile acids: The good, the bad, and the ugly. News Physiol. Sci..

[3] Begley M., Gahan C.G., Hill C. (2005). The interaction between bacteria and bile. FEMS Microbiol. Rev..

[4] Hofmann A.F. (2007). Biliary secretion and excretion in health and disease: Current concepts. Ann. Hepatol..

[5] Hofmann A.F. (1999). The continuing importance of bile acids in liver and intestinal disease. Arch. Intern. Med..

[6] Verdier J., Luedde T., Sellge G. (2015). Biliary mucosal barrier and microbiome. Viszeralmedizin.

[7] Miyake Y., Yamamoto K. (2013). Role of gut microbiota in liver diseases. Hepatol. Res..

[8] Pflughoeft K.J., Versalovic J. (2012). Human microbiome in health and disease. Annu. Rev. Pathol..

[9] Gerard P. (2013). Metabolism of cholesterol and bile acids by the gut microbiota. Pathogens.

[10] Brestoff J.R., Artis D. (2013). Commensal bacteria at the interface of host metabolism and the immune system. Nat. Immunol..

[11] Ridlon J.M., Kang D.J., Hylemon P.B., Bajaj J.S. (2014). Bile acids and the gut microbiome. Curr. Opin. Gastroenterol..

[12] Bortolini O., Medici A., Poli S. (1997). Biotransformations on steroid nucleus of bile acids. Steroids.

[13] David L.A., Maurice C.F., Carmody R.N., Gootenberg D.B., Button J.E., Wolfe B.E., Ling A.V., Devlin A.S., Varma Y., Fischbach M.A. (2014). Diet rapidly and reproducibly alters the human gut microbiome. Nature.

[14] Ridlon J.M., Kang D.J., Hylemon P.B. (2006). Bile salt biotransformations by human intestinal bacteria. J. Lipid Res..

[15] Lee J.Y., Arai H., Nakamura Y., Fukiya S., Wada M., Yokota A. (2013). Contribution of the 7β-hydroxysteroid dehydrogenase from *Ruminococcus gnavus* N53 to ursodeoxycholic acid formation in the human colon. J. Lipid Res..

[16] Jones B.V., Begley M., Hill C., Gahan C.G., Marchesi J.R. (2008). Functional and comparative metagenomic analysis of bile salt hydrolase activity in the human gut microbiome. Proc. Natl. Acad. Sci. USA.

[17] Joyce S.A., Shanahan F., Hill C., Gahan C.G. (2014). Bacterial bile salt hydrolase in host metabolism: Potential for influencing gastrointestinal microbe-host crosstalk. Gut Microbes.

[18] Begley M., Hill C., Gahan C.G. (2006). Bile salt hydrolase activity in probiotics. Appl. Environ. Microbiol..

[19] Lin J. (2014). Antibiotic growth promoters enhance animal production by targeting intestinal bile salt hydrolase and its producers. Front. Microbiol..

[20] Smith K., Zeng X., Lin J. (2014). Discovery of bile salt hydrolase inhibitors using an efficient high-throughput screening system. PLoS ONE.

[21] Dambekodi P.C., Gilliland S.E. (1998). Incorporation of cholesterol into the cellular membrane of *Bifidobacterium longum*. J. Dairy Sci..

[22] Taranto M.P., Fernandez Murga M.L., Lorca G., de Valdez G.F. (2003). Bile salts and cholesterol induce changes in the lipid cell membrane of *Lactobacillus reuteri*. J. Appl. Microbiol..

[23] Gylling H., Farkkila M., Vuoristo M., Miettinen T.A. (1995). Metabolism of cholesterol and low- and high-density lipoproteins in primary biliary cirrhosis: Cholesterol absorption and synthesis related to lipoprotein levels and their kinetics. Hepatology.

[24] Del Puppo M., Galli Kienle M., Crosignani A., Petroni M.L., Amati B., Zuin M., Podda M. (2001). Cholesterol metabolism in primary biliary cirrhosis during simvastatin and UDCA administration. J. Lipid Res..

[25] Kowdley K.V. (1998). Lipids and lipid-activated vitamins in chronic cholestatic diseases. Clin. Liver Dis..

[26] Vierling J.M. (2001). Animal models for primary sclerosing cholangitis. Best Pract. Res. Clin. Gastroenterol..

[27] Fickert P., Fuchsbichler A., Wagner M., Zollner G., Kaser A., Tilg H., Krause R., Lammert F., Langner C., Zatloukal K. (2004). Regurgitation of bile acids from leaky bile ducts causes sclerosing cholangitis in *Mdr2* (*Abcb4*) knockout mice. Gastroenterology.

[28] Jones M.L., Martoni C.J., Prakash S. (2012). Cholesterol lowering and inhibition of sterol absorption by Lactobacillus reuteri NCIMB 30242: A randomized controlled trial. Eur. J. Clin. Nutr..

[29] Degirolamo C., Rainaldi S., Bovenga F., Murzilli S., Moschetta A. (2014). Microbiota modification with probiotics induces hepatic bile acid synthesis via downregulation of the Fxr-Fgf15 axis in mice. Cell Rep..

[30] Hofmann A.F., Eckmann L. (2006). How bile acids confer gut mucosal protection against bacteria. Proc. Natl. Acad. Sci. USA.

[31] Merritt M.E., Donaldson J.R. (2009). Effect of bile salts on the DNA and membrane integrity of enteric bacteria. J. Med. Microbiol..

[32] Wahlstrom A., Sayin S.I., Marschall H.U., Backhed F. (2016). Intestinal crosstalk between bile acids and microbiota and its impact on host metabolism. Cell Metab..

[33] Weingarden A.R., Chen C., Bobr A., Yao D., Lu Y., Nelson V.M., Sadowsky M.J., Khoruts A. (2014). Microbiota transplantation restores normal fecal bile acid composition in recurrent *Clostridium difficile* infection. Am. J. Physiol. Gastrointest. Liver Physiol..

[34] Bauer T.M., Steinbruckner B., Brinkmann F.E., Ditzen A.K., Schwacha H., Aponte J.J., Pelz K., Kist M., Blum H.E. (2001). Small intestinal bacterial overgrowth in patients with cirrhosis: Prevalence and relation with spontaneous bacterial peritonitis. Am. J. Gastroenterol..

[35] Lorenzo-Zuniga V., Bartoli R., Planas R., Hofmann A.F., Vinado B., Hagey L.R., Hernandez J.M., Mane J., Alvarez M.A., Ausina V. (2003). Oral bile acids reduce bacterial overgrowth, bacterial translocation, and endotoxemia in cirrhotic rats. Hepatology.

[36] Slocum M.M., Sittig K.M., Specian R.D., Deitch E.A. (1992). Absence of intestinal bile promotes bacterial translocation. Am. Surg..

[37] Ding J.W., Andersson R., Soltesz V., Willen R., Bengmark S. (1993). The role of bile and bile acids in bacterial translocation in obstructive jaundice in rats. Eur. Surg. Res..

[38] Binder H.J., Filburn B., Floch M. (1975). Bile acid inhibition of intestinal anaerobic organisms. Am. J. Clin. Nutr..

[39] Sung J.Y., Shaffer E.A., Costerton J.W. (1993). Antibacterial activity of bile salts against common biliary pathogens. Effects of hydrophobicity of the molecule and in the presence of phospholipids. Dig. Dis. Sci..

[40] Nieman C. (1954). Influence of trace amounts of fatty acids on the growth of microorganisms. Bacteriol. Rev..

[41] Zheng C.J., Yoo J.S., Lee T.G., Cho H.Y., Kim Y.H., Kim W.G. (2005). Fatty acid synthesis is a target for antibacterial activity of unsaturated fatty acids. FEBS Lett..

[42] Brook I. (1989). Aerobic and anaerobic microbiology of biliary tract disease. J. Clin. Microbiol..

[43] Carpenter H.A. (1998). Bacterial and parasitic cholangitis. Mayo Clin. Proc..

[44] Ganzle M.G., Hertel C., van der Vossen J.M., Hammes W.P. (1999). Effect of bacteriocin-producing lactobacilli on the survival of *Escherichia coli* and *Listeria* in a dynamic model of the stomach and the small intestine. Int. J. Food Microbiol..

[45] Fox J.G., Yan L.L., Dewhirst F.E., Paster B.J., Shames B., Murphy J.C., Hayward A., Belcher J.C., Mendes E.N. (1995). *Helicobacter bilis* sp. nov., a novel *Helicobacter* species isolated from bile, livers, and intestines of aged, inbred mice. J. Clin. Microbiol..

[46] Hirai Y. (1999). The interaction of bile acids and *Helicobacter pylori*. J. Gastroenterol..

[47] Daniels J.A., Torbenson M., Anders R.A., Boitnott J.K. (2009). Immunostaining of plasma cells in primary biliary cirrhosis. Am. J. Clin. Pathol..

[48] Feizi T. (1968). Immunoglobulins in chronic liver disease. Gut.

[49] Moreira R.K., Revetta F., Koehler E., Washington M.K. (2010). Diagnostic utility of IgG and IgM immunohistochemistry in autoimmune liver disease. World J. Gastroenterol..

[50] Burroughs A.K., Rosenstein I.J., Epstein O., Hamilton-Miller J.M., Brumfitt W., Sherlock S. (1984). Bacteriuria and primary biliary cirrhosis. Gut.

[51] Butler P., Valle F., Hamilton-Miller J.M., Brumfitt W., Baum H., Burroughs A.K. (1993). M2 mitochondrial antibodies and urinary rough mutant bacteria in patients with primary biliary cirrhosis and in patients with recurrent bacteriuria. J. Hepatol..

[52] Bogdanos D.P., Baum H., Butler P., Rigopoulou E.I., Davies E.T., Ma Y., Burroughs A.K., Vergani D. (2003). Association between the primary biliary cirrhosis specific anti-sp100 antibodies and recurrent urinary tract infection. Dig. Liver Dis..

[53] Gershwin M.E., Selmi C., Worman H.J., Gold E.B., Watnik M., Utts J., Lindor K.D., Kaplan M.M., Vierling J.M., the USA PBC Epidemiology Group (2005). Risk factors and comorbidities in primary biliary cirrhosis: A controlled interview-based study of 1032 patients. Hepatology.

[54] Burroughs A.K., Butler P., Sternberg M.J., Baum H. (1992). Molecular mimicry in liver disease. Nature.

[55] Corpechot C., Chretien Y., Chazouilleres O., Poupon R. (2010). Demographic, lifestyle, medical and familial factors associated with primary biliary cirrhosis. J. Hepatol..

[56] Eaton J.E., Talwalkar J.A., Lazaridis K.N., Gores G.J., Lindor K.D. (2013). Pathogenesis of primary sclerosing cholangitis and advances in diagnosis and management. Gastroenterology.

[57] Loftus E.V., Sandborn W.J., Lindor K.D., Larusso N.F. (1997). Interactions between chronic liver disease and inflammatory bowel disease. Inflamm. Bowel Dis..

[58] Abdulkarim A.S., Petrovic L.M., Kim W.R., Angulo P., Lloyd R.V., Lindor K.D. (2004). Primary biliary cirrhosis: An infectious disease caused by Chlamydia pneumoniae?. J. Hepatol..

[59] Hopf U., Moller B., Stemerowicz R., Lobeck H., Rodloff A., Freudenberg M., Galanos C., Huhn D. (1989). Relation between *Escherichia coli* R(rough)-forms in gut, lipid A in liver, and primary biliary cirrhosis. Lancet.

[60] Smyk D.S., Rigopoulou E.I., Bogdanos D.P. (2013). Potential Roles for infectious agents in the pathophysiology of primary biliary cirrhosis: What’s new?. Curr. Infect. Dis. Rep..

[61] Nilsson H.O., Taneera J., Castedal M., Glatz E., Olsson R., Wadstrom T. (2000). Identification of *Helicobacter pylori* and other *Helicobacter* species by PCR, hybridization, and partial DNA sequencing in human liver samples from patients with primary sclerosing cholangitis or primary biliary cirrhosis. J. Clin. Microbiol..

[62] Nilsson H.O., Taneera J., Castedal M., Wadstrom T., Olsson R. (2000). Infectious agents and primary biliary cirrhosis. J. Hepatol..

[63] Xu L., Shen Z., Guo L., Fodera B., Keogh A., Joplin R., O’Donnell B., Aitken J., Carman W., Neuberger J. (2003). Does a β retrovirus infection trigger primary biliary cirrhosis?. Proc. Natl. Acad. Sci. USA.

[64] Olsson R., Bjornsson E., Backman L., Friman S., Hockerstedt K., Kaijser B., Olausson M. (1998). Bile duct bacterial isolates in primary sclerosing cholangitis: A study of explanted livers. J. Hepatol..

[65] Pollheimer M.J., Halilbasic E., Fickert P., Trauner M. (2011). Pathogenesis of primary sclerosing cholangitis. Best Pract. Res. Clin. Gastroenterol..

[66] Fussey S.P., Ali S.T., Guest J.R., James O.F., Bassendine M.F., Yeaman S.J. (1990). Reactivity of primary biliary cirrhosis sera with *Escherichia coli* dihydrolipoamide acetyltransferase (E2p): Characterization of the main immunogenic region. Proc. Natl. Acad. Sci. USA.

[67] Selmi C., Balkwill D.L., Invernizzi P., Ansari A.A., Coppel R.L., Podda M., Leung P.S., Kenny T.P., van de Water J., Nantz M.H. (2003). Patients with primary biliary cirrhosis react against a ubiquitous xenobiotic-metabolizing bacterium. Hepatology.

[68] Bogdanos D.P., Baum H., Grasso A., Okamoto M., Butler P., Ma Y., Rigopoulou E., Montalto P., Davies E.T., Burroughs A.K. (2004). Microbial mimics are major targets of crossreactivity with human pyruvate dehydrogenase in primary biliary cirrhosis. J. Hepatol..

[69] Bogdanos D.P., Baum H., Okamoto M., Montalto P., Sharma U.C., Rigopoulou E.I., Vlachogiannakos J., Ma Y., Burroughs A.K., Vergani D. (2005). Primary biliary cirrhosis is characterized by IgG3 antibodies cross-reactive with the major mitochondrial autoepitope and its *Lactobacillus* mimic. Hepatology.

[70] Bogdanos D.P., Pares A., Baum H., Caballeria L., Rigopoulou E.I., Ma Y., Burroughs A.K., Rodes J., Vergani D. (2004). Disease-specific cross-reactivity between mimicking peptides of heat shock protein of *Mycobacterium gordonae* and dominant epitope of E2 subunit of pyruvate dehydrogenase is common in Spanish but not British patients with primary biliary cirrhosis. J. Autoimmun..

[71] Padgett K.A., Selmi C., Kenny T.P., Leung P.S., Balkwill D.L., Ansari A.A., Coppel R.L., Gershwin M.E. (2005). Phylogenetic and immunological definition of four lipoylated proteins from *Novosphingobium aromaticivorans*, implications for primary biliary cirrhosis. J. Autoimmun..

[72] Kaplan M.M., Gershwin M.E. (2005). Primary biliary cirrhosis. N. Engl. J. Med..

[73] Baum H., Bogdanos D.P., Vergani D. (2001). Antibodies to Clp protease in primary biliary cirrhosis: Possible role of a mimicking T-cell epitope. J. Hepatol..

[74] Barbeau J., Tanguay R., Faucher E., Avezard C., Trudel L., Cote L., Prevost A.P. (1996). Multiparametric analysis of waterline contamination in dental units. Appl. Environ. Microbiol..

[75] Cavicchioli R., Fegatella F., Ostrowski M., Eguchi M., Gottschal J. (1999). Sphingomonads from marine environments. J. Ind. Microbiol. Biotechnol..

[76] Brodie E.L., DeSantis T.Z., Parker J.P., Zubietta I.X., Piceno Y.M., Andersen G.L. (2007). Urban aerosols harbor diverse and dynamic bacterial populations. Proc. Natl. Acad. Sci. USA.

[77] Mattner J., Savage P.B., Leung P., Oertelt S.S., Wang V., Trivedi O., Scanlon S.T., Pendem K., Teyton L., Hart J. (2008). Liver autoimmunity triggered by microbial activation of natural killer T cells. Cell Host Microbe.

[78] Mohammed J.P., Fusakio M.E., Rainbow D.B., Moule C., Fraser H.I., Clark J., Todd J.A., Peterson L.B., Savage P.B., Wills-Karp M. (2011). Identification of *Cd101* as a susceptibility gene for *Novosphingobium aromaticivorans*-induced liver autoimmunity. J. Immunol..

[79] Mattner J. (2013). Natural killer T (NKT) cells in autoimmune hepatitis. Curr. Opin. Immunol..

[80] Shuttleworth K.L., Sung J., Kim E., Cerniglia C.E. (2000). Physiological and genetic comparison of two aromatic hydrocarbon-degrading *Sphingomonas* strains. Mol. Cells.

[81] Shi T., Fredrickson J.K., Balkwill D.L. (2001). Biodegradation of polycyclic aromatic hydrocarbons by *Sphingomonas* strains isolated from the terrestrial subsurface. J. Ind. Microbiol. Biotechnol..

[82] Pinyakong O., Habe H., Omori T. (2003). The unique aromatic catabolic genes in sphingomonads degrading polycyclic aromatic hydrocarbons (PAHs). J. Gen. Appl. Microbiol..

[83] Zhang H., Carbone M., Lleo A., Invernizzi P. (2015). Geoepidemiology, genetic and environmental risk factors for PBC. Dig. Dis..

[84] Metcalf J.V., Mitchison H.C., Palmer J.M., Jones D.E., Bassendine M.F., James O.F. (1996). Natural history of early primary biliary cirrhosis. Lancet.

[85] Trivedi P.J., Adams D.H. (2013). Mucosal immunity in liver autoimmunity: A comprehensive review. J. Autoimmun..

[86] Katt J., Schwinge D., Schoknecht T., Quaas A., Sobottka I., Burandt E., Becker C., Neurath M.F., Lohse A.W., Herkel J. (2013). Increased T helper type 17 response to pathogen stimulation in patients with primary sclerosing cholangitis. Hepatology.

[87] Folseraas T., Melum E., Rausch P., Juran B.D., Ellinghaus E., Shiryaev A., Laerdahl J.K., Ellinghaus D., Schramm C., Weismuller T.J. (2012). Extended analysis of a genome-wide association study in primary sclerosing cholangitis detects multiple novel risk loci. J. Hepatol..

[88] Hiramatsu K., Harada K., Tsuneyama K., Sasaki M., Fujita S., Hashimoto T., Kaneko S., Kobayashi K., Nakanuma Y. (2000). Amplification and sequence analysis of partial bacterial 16S ribosomal RNA gene in gallbladder bile from patients with primary biliary cirrhosis. J. Hepatol..

[89] Jimenez E., Sanchez B., Farina A., Margolles A., Rodriguez J.M. (2014). Characterization of the bile and gall bladder microbiota of healthy pigs. Microbiol. Open.

[90] Packey C.D., Sartor R.B. (2009). Commensal bacteria, traditional and opportunistic pathogens, dysbiosis and bacterial killing in inflammatory bowel diseases. Curr. Opin. Infect. Dis..

[91] Reiff C., Kelly D. (2010). Inflammatory bowel disease, gut bacteria and probiotic therapy. Int. J. Med. Microbiol..

[92] Tabibian J.H., Talwalkar J.A., Lindor K.D. (2013). Role of the microbiota and antibiotics in primary sclerosing cholangitis. BioMed Res. Int..

[93] Lichtman S.N., Keku J., Clark R.L., Schwab J.H., Sartor R.B. (1991). Biliary tract disease in rats with experimental small bowel bacterial overgrowth. Hepatology.

[94] Lichtman S.N., Okoruwa E.E., Keku J., Schwab J.H., Sartor R.B. (1992). Degradation of endogenous bacterial cell wall polymers by the muralytic enzyme mutanolysin prevents hepatobiliary injury in genetically susceptible rats with experimental intestinal bacterial overgrowth. J. Clin. Investig..

[95] Rossen N.G., Fuentes S., Boonstra K., D’Haens G.R., Heilig H.G., Zoetendal E.G., de Vos W.M., Ponsioen C.Y. (2015). The mucosa-associated microbiota of PSC patients is characterized by low diversity and low abundance of uncultured *Clostridiales* II. J. Crohns Colitis.

[96] Quraishi M.N., Sergeant M., Kay G., Iqbal T., Chan J., Constantinidou C., Trivedi P., Ferguson J., Adams D.H., Pallen M. (2016). The gut-adherent microbiota of PSC-IBD is distinct to that of IBD. Gut.

[97] Sabino J., Vieira-Silva S., Machiels K., Joossens M., Falony G., Ballet V., Ferrante M., van Assche G., van der Merwe S., Vermeire S. (2016). Primary sclerosing cholangitis is characterised by intestinal dysbiosis independent from IBD. Gut.

[98] Rupp C., Bode K.A., Chahoud F., Wannhoff A., Friedrich K., Weiss K.H., Sauer P., Stremmel W., Gotthardt D.N. (2014). Risk factors and outcome in patients with primary sclerosing cholangitis with persistent biliary candidiasis. BMC Infect. Dis..

[99] Rausch P., Rehman A., Kunzel S., Hasler R., Ott S.J., Schreiber S., Rosenstiel P., Franke A., Baines J.F. (2011). Colonic mucosa-associated microbiota is influenced by an interaction of Crohn disease and FUT2 (Secretor) genotype. Proc. Natl. Acad. Sci. USA.

[100] Pickard J.M., Maurice C.F., Kinnebrew M.A., Abt M.C., Schenten D., Golovkina T.V., Bogatyrev S.R., Ismagilov R.F., Pamer E.G., Turnbaugh P.J. (2014). Rapid fucosylation of intestinal epithelium sustains host-commensal symbiosis in sickness. Nature.

[101] Terjung B., Sohne J., Lechtenberg B., Gottwein J., Muennich M., Herzog V., Mahler M., Sauerbruch T., Spengler U. (2010). p-ANCAs in autoimmune liver disorders recognise human β-tubulin isotype 5 and cross-react with microbial protein FtsZ. Gut.

[102] Karrar A., Broome U., Sodergren T., Jaksch M., Bergquist A., Bjornstedt M., Sumitran-Holgersson S. (2007). Biliary epithelial cell antibodies link adaptive and innate immune responses in primary sclerosing cholangitis. Gastroenterology.

[103] Tabibian J.H., O’Hara S.P., Trussoni C.E., Tietz P.S., Splinter P.L., Mounajjed T., Hagey L.R., LaRusso N.F. (2016). Absence of the intestinal microbiota exacerbates hepatobiliary disease in a murine model of primary sclerosing cholangitis. Hepatology.

[104] Swann J.R., Want E.J., Geier F.M., Spagou K., Wilson I.D., Sidaway J.E., Nicholson J.K., Holmes E. (2011). Systemic gut microbial modulation of bile acid metabolism in host tissue compartments. Proc. Natl. Acad. Sci. USA.

[105] Cox K.L., Cox K.M. (1998). Oral vancomycin: Treatment of primary sclerosing cholangitis in children with inflammatory bowel disease. J. Pediatr. Gastroenterol. Nutr..

[106] Farkkila M., Karvonen A.L., Nurmi H., Nuutinen H., Taavitsainen M., Pikkarainen P., Karkkainen P. (2004). Metronidazole and ursodeoxycholic acid for primary sclerosing cholangitis: A randomized placebo-controlled trial. Hepatology.

[107] Davies Y.K., Cox K.M., Abdullah B.A., Safta A., Terry A.B., Cox K.L. (2008). Long-term treatment of primary sclerosing cholangitis in children with oral vancomycin: An immunomodulating antibiotic. J. Pediatr. Gastroenterol. Nutr..

[108] Silveira M.G., Torok N.J., Gossard A.A., Keach J.C., Jorgensen R.A., Petz J.L., Lindor K.D. (2009). Minocycline in the treatment of patients with primary sclerosing cholangitis: Results of a pilot study. Am. J. Gastroenterol..

[109] Elfaki D.A., Lindor K.D. (2011). Antibiotics for the treatment of primary sclerosing cholangitis. Am. J. Ther..

[110] Tabibian J.H., Weeding E., Jorgensen R.A., Petz J.L., Keach J.C., Talwalkar J.A., Lindor K.D. (2013). Randomised clinical trial: Vancomycin or metronidazole in patients with primary sclerosing cholangitis—A pilot study. Aliment. Pharmacol. Ther..

[111] Goode E.C., Rushbrook S.M. (2016). A review of the medical treatment of primary sclerosing cholangitis in the 21st century. Ther. Adv. Chronic Dis..

[112] Cho I., Blaser M.J. (2012). The human microbiome: At the interface of health and disease. Nat. Rev. Genet..

[113] Cho I., Yamanishi S., Cox L., Methe B.A., Zavadil J., Li K., Gao Z., Mahana D., Raju K., Teitler I. (2012). Antibiotics in early life alter the murine colonic microbiome and adiposity. Nature.

[114] Dancer S.J. (2004). How antibiotics can make us sick: The less obvious adverse effects of antimicrobial chemotherapy. Lancet Infect. Dis..

[115] Hempel S., Newberry S.J., Maher A.R., Wang Z., Miles J.N., Shanman R., Johnsen B., Shekelle P.G. (2012). Probiotics for the prevention and treatment of antibiotic-associated diarrhea: A systematic review and meta-analysis. JAMA.

[116] Power S.E., O’Toole P.W., Stanton C., Ross R.P., Fitzgerald G.F. (2014). Intestinal microbiota, diet and health. Br. J. Nutr..

[117] Mogg G.A., Keighley M.R., Burdon D.W., Alexander-Williams J., Youngs D., Johnson M., Bentley S., George R.H. (1979). Antibiotic-associated colitis—A review of 66 cases. Br. J. Surg..

[118] Cunha B.A. (2001). Antibiotic side effects. Med. Clin. N. Am..

[119] Bartlett J.G. (2002). Antibiotic-associated diarrhea. N. Engl. J. Med..

[120] Jernberg C., Lofmark S., Edlund C., Jansson J.K. (2007). Long-term ecological impacts of antibiotic administration on the human intestinal microbiota. ISME J..

[121] Dethlefsen L., Huse S., Sogin M.L., Relman D.A. (2008). The pervasive effects of an antibiotic on the human gut microbiota, as revealed by deep 16S rRNA sequencing. PLoS Biol..

[122] Antonopoulos D.A., Huse S.M., Morrison H.G., Schmidt T.M., Sogin M.L., Young V.B. (2009). Reproducible community dynamics of the gastrointestinal microbiota following antibiotic perturbation. Infect. Immun..

[123] Hill D.A., Hoffmann C., Abt M.C., Du Y., Kobuley D., Kirn T.J., Bushman F.D., Artis D. (2010). Metagenomic analyses reveal antibiotic-induced temporal and spatial changes in intestinal microbiota with associated alterations in immune cell homeostasis. Mucosal Immunol..

[124] Ubeda C., Taur Y., Jenq R.R., Equinda M.J., Son T., Samstein M., Viale A., Socci N.D., van den Brink M.R., Kamboj M. (2010). Vancomycin-resistant *Enterococcus* domination of intestinal microbiota is enabled by antibiotic treatment in mice and precedes bloodstream invasion in humans. J. Clin. Investig..

[125] Hviid A., Svanstrom H., Frisch M. (2011). Antibiotic use and inflammatory bowel diseases in childhood. Gut.

[126] Hill D.A., Siracusa M.C., Abt M.C., Kim B.S., Kobuley D., Kubo M., Kambayashi T., Larosa D.F., Renner E.D., Orange J.S. (2012). Commensal bacteria-derived signals regulate basophil hematopoiesis and allergic inflammation. Nat. Med..

[127] Russell S.L., Gold M.J., Hartmann M., Willing B.P., Thorson L., Wlodarska M., Gill N., Blanchet M.R., Mohn W.W., McNagny K.M. (2012). Early life antibiotic-driven changes in microbiota enhance susceptibility to allergic asthma. EMBO Rep..

[128] Greer R.L., Morgun A., Shulzhenko N. (2013). Bridging immunity and lipid metabolism by gut microbiota. J. Allergy Clin. Immunol..

[129] Cox L.M., Yamanishi S., Sohn J., Alekseyenko A.V., Leung J.M., Cho I., Kim S.G., Li H., Gao Z., Mahana D. (2014). Altering the intestinal microbiota during a critical developmental window has lasting metabolic consequences. Cell.

[130] Weingarden A.R., Chen C., Zhang N., Graiziger C.T., Dosa P.I., Steer C.J., Shaughnessy M.K., Johnson J.R., Sadowsky M.J., Khoruts A. (2016). Ursodeoxycholic acid inhibits clostridium difficile spore germination and vegetative growth, and prevents the recurrence of ileal pouchitis associated with the infection. J. Clin. Gastroenterol..

[131] Islam K.B., Fukiya S., Hagio M., Fujii N., Ishizuka S., Ooka T., Ogura Y., Hayashi T., Yokota A. (2011). Bile acid is a host factor that regulates the composition of the cecal microbiota in rats. Gastroenterology.

[132] Yokota A., Fukiya S., Islam K.B., Ooka T., Ogura Y., Hayashi T., Hagio M., Ishizuka S. (2012). Is bile acid a determinant of the gut microbiota on a high-fat diet?. Gut Microbes.

